# Waveform Analysis of UWB GPR Antennas

**DOI:** 10.3390/s90301454

**Published:** 2009-03-03

**Authors:** Fernando I. Rial, Henrique Lorenzo, Manuel Pereira, Julia Armesto

**Affiliations:** Natural Resources & Environmental Engineering, EUET Forestal. University of Vigo. Campus A Xunqueira s/n. 36005 Pontevedra, Spain.

**Keywords:** Bow-tie antenna, source wavelet, time domain, frequency domain

## Abstract

Ground Penetrating Radar (GPR) systems fall into the category of ultra-wideband (UWB) devices. Most GPR equipment covers a frequency range between an octave and a decade by using short-time pulses. Each signal recorded by a GPR gathers a temporal log of attenuated and distorted versions of these pulses (due to the effect of the propagation medium) plus possible electromagnetic interferences and noise. In order to make a good interpretation of this data and extract the most possible information during processing, a deep knowledge of the wavelet emitted by the antennas is essential. Moreover, some advanced processing techniques require specific knowledge of this signal to obtain satisfactory results. In this work, we carried out a series of tests in order to determine the source wavelet emitted by a ground-coupled antenna with a 500 MHz central frequency.

## 1. Introduction

According to [1], ultra-wideband (UWB) technology in radars basically extends into three different areas according to their application range: short, medium, and long range. Leading components within each of these areas include: Ground-or-Surface Penetrating Radars (GPR), Terrain surveillance radars, and Synthetic Aperture Radars (SAR), respectively. At the moment the most widely used UWB radar is GPR, which has been commercialized by more than a dozen companies around the world [2].

Due to the widespread proliferation of electromagnetic devices, the radio spectrum has become a limited resource, so that the “broad intrusion” of the GPR signals in an already crowded spectrum has recently resulted in proposals that establish rules and regulations regarding the use and characteristics of these devices [3, 4].

The main advantage of using UWB technology for surface radars is determined by the need for high vertical resolution (also known as down-range or depth resolution), which is closely related to the radar operational bandwidth [5]:

(1) $$\Delta v\;=\;\frac{c}{2\times \Delta f}$$

where Δv is the vertical resolution of the system, c is the velocity of an electromagnetic signal in vacuum, and Δf is the bandwidth used by the system.

Due to the use of UWB technology, remote sensing devices known as radar were divided into two broad categories: radars (themselves) and sensors. In terms of its design, the main difference between them lies in the receiver chain. Radars usually have amplitude peak detectors whereas sensors preserve the signal for further analysis [6], which can provide valuable additional information. In this sense, current GPR equipment should be reclassified as sensors.

Most GPR equipment meets the required bandwidth specifications for a UWB device using very short-time pulses or impulses transmitted at baseband (without an intermediate carrying frequency). That is why such GPR systems are called time domain systems. Each trace obtained by the radar is composed of distorted and attenuated versions (as a result of the propagation medium) of the pulse emitted by the antennas. Part of the antennas construction is still a process done mainly by hand and even antennas from the same company and with the same nominal frequency present, for instance, slight differences in terms of this emitted wavelet or in the radiation pattern. It is also important to notice that since GPR antennas usually consist of two dipoles – one for transmission and another for reception – the effective wavelet recorded will be dependent on the characteristics of both antennas, not just the transmitter.

Therefore, in order to make a good interpretation of the GPR records and extract as much information as possible from the signal recorded during processing, a deep knowledge of the type of emission used is essential because the characteristics of the detected reflections (length and shape of the reflected pulse, overlapping of constructive or destructive reflections, etc.) directly depend on the characteristics of the wavelet emitted by the antennas [7, 8]. In addition, advanced processing techniques such as deconvolution or specific algorithms for target recognition require specific knowledge of this signal for proper operation.

Within the field of numerical simulation, it is also useful to work with the real source wavelet of the system. The goal of the simulation is to obtain a synthetic record very similar to that obtained in the field, which could aid in data interpretation. To provide practical results, modulation schemes in computer simulators should be able to incorporate, in addition to real antenna configurations and appropriate descriptions of the material properties, a precise model of the signal emitted by the antennas [9]. However, the manner in which these modelling programs make use of this wavelet should be considered since there are several methods, some of which are somewhat complex to implement if the intention is to use a user-defined wavelet.

With all this in mind and an aim to deepen the knowledge of our system, we have designed a set of experiments with a relatively simple methodology so that they can be performed without specialized instrumentation. A basic knowledge of the radar operation and some additional simple tools are enough for a user to carry out the tests to study the particular characteristics, in the time and frequency domains, of the wavelet emitted by the antennas.

The characteristics of the emitted signal in the time domain are related to pulse shape, number of cycles or semi-cycles composing the pulse, temporal length, and geometric expansion. On the other hand, frequency analysis identifies characteristic parameters of the signal in this domain, such as the frequency spectrum used, relative power per spectral line, antenna central frequency, etc. Additionally, because the antenna consists of two dipoles, the direct signal between both is always present in any radar trace, so it is also interesting to study it in detail. For the realization of the proposed methodologies, a spacious laboratory has proven to be adequate to test medium- to high-frequency antennas as evaluated in this work.

## 2. Previous Considerations

### 2.1. Characteristics of the equipment

**Figure 1.**
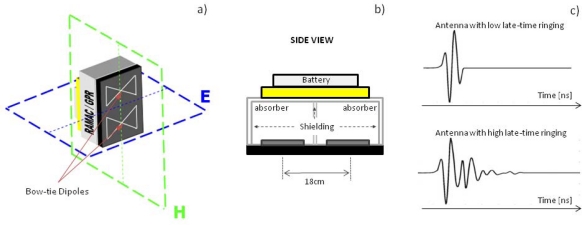
(a) Dipole arrangement inside the antenna and principal radiation planes. (b) Antenna′s internal view. (c) Differences between pulses with low and high late-time ringing.

The equipment evaluated in this work is a RAMAC/GPR CUII (MALA Geoscience) and a shielded ground-coupled antenna with a nominal central frequency of 500 MHz. The antenna consists of two bow-tie dipoles [Figure 1(a)] protected by a shell as a shielding designed to isolate the antenna from external interference and possible reflections from objects on the surface. An absorbent material covers the inner walls of the antenna in order to eliminate internal reflections [Figure 1(b)]. The exact size and shape of the bow-tie dipoles inside the antenna is unknown, but the internal distance between transmitter and receiver dipoles is provided by the manufacturer as being 18cm.

As mentioned earlier, radar resolution can be compromised if the impulse response of the antenna is significantly extended, so for these systems, antennas with low late-time ringing are required [Figure 1(c)]. This necessitates a clean shape of the radiated pulse to avoid overlapping between close targets, which is why most of the manufactured bow-tie antennas are designed and created following a profile of increasing resistance as it approaches its ends (Wu-King profile) to improve temporal resolution. On the other hand, this design results in a cost in efficiency of the antenna, and different alternatives are being investigated [10].

Due to the use of two dipoles, one for transmission and the other for reception, the direct signal between both is always registered in any trace. *A priori*, this signal might be considered a possible approximation of the emitted pulse, but one must take into account both the proximity between the two dipoles and their arrangement inside the antenna [not opposite; Figure 1(a)]; on the other hand, due to the antenna shielding, it is possible that some internal reflection can reach the receiver almost simultaneously in spite of the absorbent material, substantially varying the characteristics of the signal recorded in comparison with the emitted wavelet.

### 2.2. Influence of the medium

Ground-coupled antennas are designed to be located near the surface so that the power radiated into the medium under study is the maximum possible. When an antenna is located on an interface, the two most important factors that are affected by the presence of this medium (usually a lossy dielectric) are the antenna current distribution and radiation pattern [11].

On the one hand, the current velocity along the antenna varies, thus changing the resonance frequency. This velocity will have a value between the velocity of an electromagnetic wave in the air and in the dielectric medium. This effect can be quantified by considering that the antenna is immersed in a medium with a permittivity equal to the average between the two materials [12]. A delay in the velocity means a lower frequency and, as a consequence, the pulse spectrum is shifted to lower frequencies. On the other hand, the medium generally acts as a low pass filter in such a way that it modifies the spectrum of the transmitted signal as a function of the medium’s electromagnetic properties.

### 2.3. DC Component

**Figure 2.**
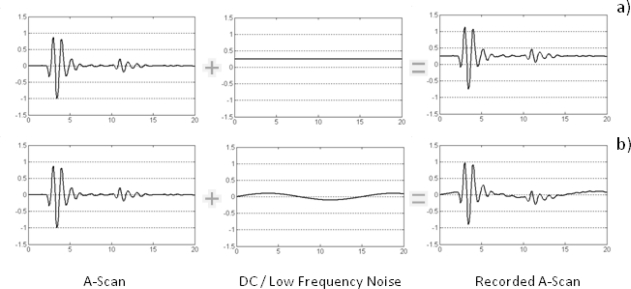
Superposition of a continuous or low frequency component (DC) to the radar signal. This component can be constant (a) or variable (b) along the trace.

Another common feature in commercialized GPR equipment is the appearance of a continuous or low frequency component (DC component) in the traces (or A-scans) recorded by the radar, so the averaged level of the signal is moved from zero amplitude to a different value (Figure 2). The appearance of this component is usually associated with both inductive phenomena and limitations on the system’s dynamic range [13].

DC-levels often vary depending on the medium under the antenna and the antenna-surface distance, so it is common for this component to differ slightly from one trace to another in a continuous profile or B-scan. The elimination of this component is a prerequisite not only visually, but also for the implementation of subsequent data processing because otherwise, the results may differ significantly from what is expected [14].

### 2.4. Geometric Attenuation

The relationship between the power transmitted and received by a radar system is given by the well-known radar range equation (or for simplicity, the radar equation or range equation) [5]:

(2) $$\frac{P_{r}}{P_{t}}=\frac{G_{t}A_{r}\sigma }{{\left(4\pi \right)}^{2}R^{4}}$$

where Gt is the gain of transmitting antenna, Ar is the effective area of the receiving antenna (antenna aperture), R is the range (target distance), and σ is the radar section.

In this equation, the geometric attenuation losses of the front wave emitted by the antenna are associated in a conventional manner with the fourth power of the inverse distance for a point reflector. If the type of reflector fits better to a line reflector (pipeline) or planar reflector (smooth interface), it is necessary to establish a correction factor in the equation of R^2^ and R^3^ instead of R^4^, respectively [12].

### 2.5. Polarity changes in the reflected signal

**Figure 3.**
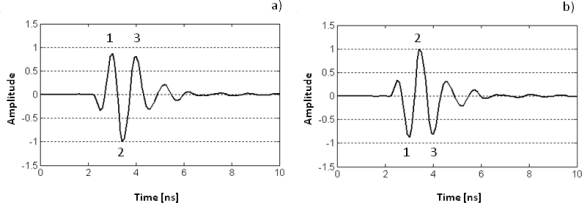
Signals (a) and (b) represent the same wavelet but with different polarities.

When a pulse emitted by an antenna bounces off an interface between two layers of different materials or on an object, a change in the signal polarity can occur. A change in polarity represents an inversion of the reflected amplitudes with respect to its original ones (Figure 3).

Changes in polarity of the reflected wavelet take place when the new medium or object has an impedance lower than the previous medium. Otherwise, the signal is reflected with the same polarity. Changes in the pulse polarity have been used by several authors as an advanced method of detecting cavities under paved areas or concrete slabs [15–17].

## 3. Methodology

**Figure 4.**
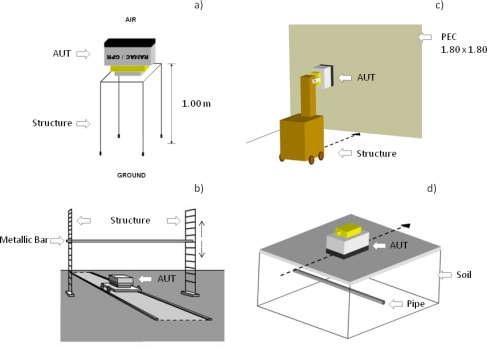
Methodologies used during the tests to obtain: (a) Direct signal between dipoles in a reflector-free environment. (b) Emitted wavelet in air reflected in a metallic bar at different distances. (c) Emitted wavelet in air reflected in a PEC at different distances. (d) Emitted wavelet in a lossy dielectric reflected in a large buried pipe.

To obtain the direct signal between dipoles, the antenna under test (AUT) was placed in an environment free of any reflectors that might overlap the registration of this signal. The direction of maximum radiation of the dipoles pointed into the air, leaving a plane containing both dipoles parallel to the ground [Figure 4(a)]. The antenna was subjected to a warm up time of 10 min before starting the tests. This warm up time consisted of keeping the equipment acquiring data triggered by time at fast speed.

A methodology often used to achieve a very approximate version of the emitted signal is to obtain a reflection of this wavelet from an object or surface [18–20]. Given its characteristics of linearity, homogeneity, isotropy, and non-dispersion, air becomes an ideal propagation medium in this case, ensuring that the waveform received is not affected by any of these effects that, to a greater or lesser extent, are present in all environments in which a study with a GPR is carried out. The main correction factor associated with this reflection is the geometric attenuation of the signal. This factor is also analyzed from the obtained results.

With this in mind, two simple tests were performed with the AUT. The first is shown in Figure 4(c), where each antenna was placed against a wall that is covered with aluminium foil because its high conductivity (σ = 3.54 • 10^7^ S/m) and reflection coefficient in air (0.99996), which is close to a perfect electric conductor (PEC). On the other hand, this ensures that the back reflection due to the wall-air interface will not overlap the first reflection. The dimensions of the PEC were 180 × 180 cm.

The scheme followed in the second test can be observed in Figure 4(b). In this case, a reflector consisting of a metallic bar 3 cm in diameter was used. As can be appreciated, the dipole’s orientation is parallel to the axis of the bar, maximizing the intensity of the reflection in the E-plane. The metallic bar is placed just in the plane of separation between the two dipoles.

In the two tests discussed (PEC and metallic bar), instead of a single measurement, a series of successive measurements was carried out with a gradual separation between the antenna and reflector. In so doing, these tests could be used to characterize both the geometric attenuation of the signal and determine the minimum distance of overlap between the direct signal and a first reflector.

Finally, one last test was conducted over a paved area where a large pipe was known to be present [Figure 4(d)]. The main goal here was to carry out a profile transversal to the longitudinal axis of the pipe in order to obtain a clear reflection of the emitted wavelet on the pipe.

## 4. Results

### 4.1. Time Domain Analysis

#### Direct Signal

**Figure 5.**
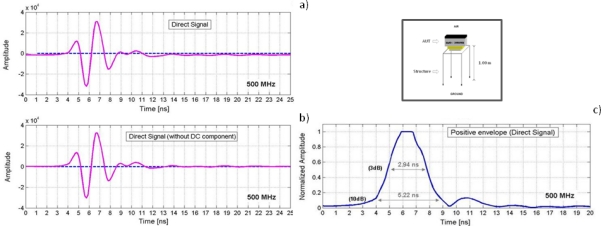
Direct Signal between dipoles obtained for the AUT. (a) Recorded A-Scan. (b) Recorded A-Scan without DC component. (c) Positive envelope of the signal

Figure 5 shows a trace (A-scan) containing the direct signal between the internal transmitter and receiver dipoles for the AUT. This has been obtained by the methodology of Figure 4(a), where the AUT was oriented towards the air in an environment free of external reflectors in order to isolate this signal. The upper left side of Figure 5(a) shows the signal as recorded, without having applied any filter or gain. It should be noted that a slight DC component was added to the obtained trace. The presence of this component in the data has been previously discussed. In the lower left side [Figure 5(b)], the same trace as before but with the average level set to zero is shown. Figure 5(c) shows the absolute value of the envelope, where the amplitude values have been normalized with respect to the maximum amplitude peak. This figure also indicates the time intervals between values of 1/2 (corresponding to 3 dB) and 1/10 (corresponding to 10 dB) with respect to the recorded maximum amplitude.

#### Reflected wavelet in air (Metallic Bar and PEC)

**Figure 6.**
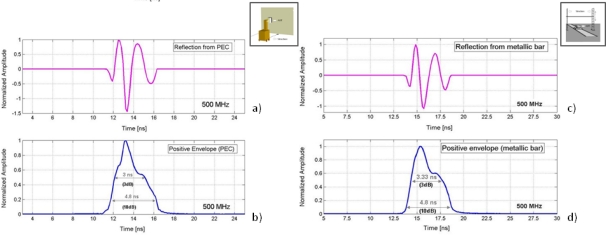
Reflected wavelet in air obtained for the AUT. (a-b) Recorded wavelet and positive envelope (PEC). (c-d) Recorded wavelet and positive envelope (metallic bar).

Figure 6 shows an example of the recorded pulse after its reflection in the metallic bar and aluminium surface according to the methodology schematized in Figures 4(b) and 4(c). It also represents the envelope of the pulse and, as in the analysis of the direct signal, some parameters concerning its duration are indicated.

**Figure 7.**
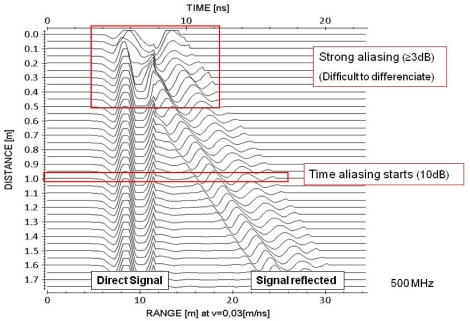
Obtained radargram where it is possible to observe the recorded wavelet after its reflection in the metallic bar at different distances.

As discussed in the methodology, for the reflection tests on the metallic bar and aluminium surface instead of a single measurement a series of successive measurements was carried out with a gradual separation between the AUT and the reflector. This allows for a study of the distance of the minimum overlap between the direct signal and the reflection from a surface. Figure 7 shows the sequence of consecutive traces in the records obtained for different separations between the metallic bar and the antenna.

The record of Figure 7, like its counterpart obtained from the PEC, allows for the determination of the 3 dB and 10 dB overlapping distances between the direct signal and a surface or object close to the antenna. The results obtained in both experiments (PEC and metal bar) show good correlation with results of 55 cm (0.94 λ) and 100 cm (1.57 λ) for 3 and 10 dB, respectively. These values represent, in general, the minimum distance between the antenna and an object or surface so that aliasing with the direct signal between dipoles does not occur.

**Figure 8.**
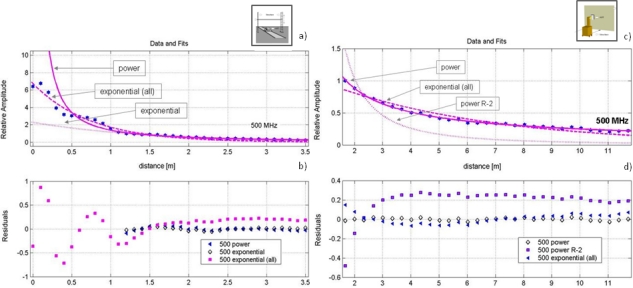
Maximum amplitude peaks of the reflected wavelet at different distances. For each methodology, three possible regression curves and their residual variations are shown. (a-b) Metallic bar. (c-d) PEC.

From the records obtained by this methodology, it is possible to study the attenuation suffered by the reflection as it is separated from the antenna. The amplitude values used in the study are the peaks of maximum amplitude of the reflections for each position. Figure 8 shows possible regression curves of the results obtained with the 500 MHz antenna.

Both the equations of the represented curves, as well as the values of the variables that allow the best fit of the obtained peaks are shown in Table 1. This table also shows the parameters related to the quality of the fit.

#### Reflected wavelet in lossy dielectric

**Figure 9.**
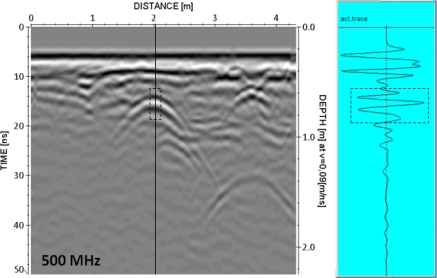
Radargram obtained in the study area. A large pipe in its central part is clearly visible. The trace from which the wavelet was obtained is marked on the radargram and shown on the left.

Concerning the tests in a medium different than air, the B-scan (continuous two-dimensional profile) obtained in the study area chosen for this purpose is shown in Figure 9. In the central part of the radargram, the presence of a pipe is notable. The rebound of the signal on the surface of this pipe is the pulse taken as an example for analysis.

**Figure 10.**
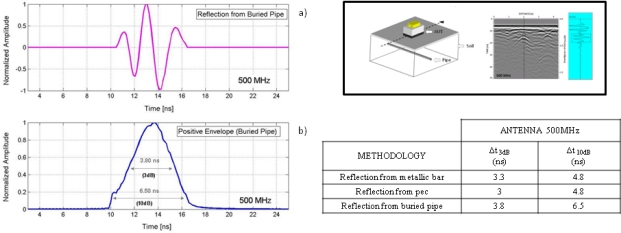
a) Wavelet obtained from the buried pipe. b) Positive envelope and time intervals between values of 1/2 (corresponding to 3 dB) and 1/10 (corresponding to 10 dB) with respect to the recorded maximum amplitude. A table with parameters related to the duration of the reflected wavelet obtained in the different experiments is also included.

As in previous cases, the signals obtained and some data concerning the characteristics in time of the pulse are shown in Figure 10. A table with parameters related to the duration of the wavelets obtained in this experiment and in the two previous ones is also shown so they can be easily compared.

**Figure 11.**
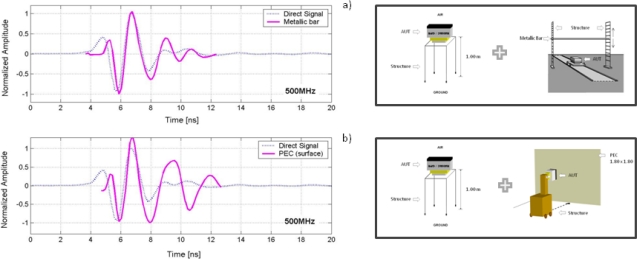
a) Wavelet obtained with the metallic bar and direct signal between dipoles. b) Wavelet obtained from the PEC and direct signal between dipoles. (Both signals were normalized and inverted in phase for comparison with the direct signal).

Figure 11 compares wavelets gained from experiments on the metal bar and the PEC with the direct signal between dipoles. The wavelets are inverted prior to comparison with the direct signal to obtain a better fit.

### 4.2. Frequency Domain Analysis

**Figure 12.**
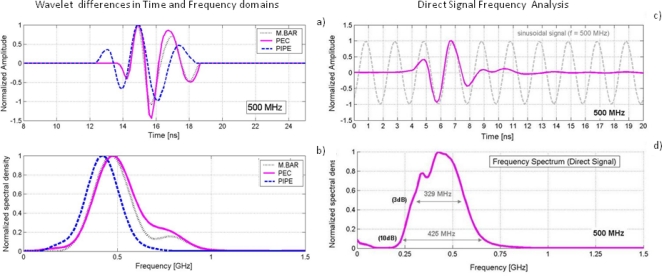
(a) Wavelet differences in the time domain. (b) Wavelet differences in the frequency domain. (c-d) Frequency analysis of the direct signal between dipoles.

Figure 12 shows a frequency analysis of the obtained signals that have been analyzed in the time domain in the previous experiments.

Figure 12(c) shows the good correlation in time domain between the direct signal and a pure frequency tone of 500 MHz. Figure 12(d) displays the power spectral density of the direct signal, where its bandwidth at the 3 and 10 dB levels have been indicated. The left side of Figure 12(a-b) shows the comparison, in the time and frequency domains, of the calculated emitted wavelets following the three different methodologies. Table 2 contains values of interest related to the frequency characterization of the effective wavelets obtained with the different methodologies.

## 5. Discussion

In this work, a methodological proposal has been presented with the main objective of determining the characteristics of the source wavelet emitted by a GPR ground-coupled antenna. In order to do that, three different tests were presented: two in air with a similar methodology but using a metallic bar in one case and an aluminium surface in another, and a test pipe buried in lossy dielectric. These results have been compared and demonstrate that it is possible to draw the following conclusions.

From the test results, it has been possible to obtain an approximation of the emitted wavelet. The analysis in time of the length and shape of the wavelets has allowed an approximate characterization of these signals. The frequency domain analysis has revealed the frequency ranges used by our antenna and its spectral power distribution. The type of methodology employed in the experiments is not a direct method, in the sense that what is obtained is not a direct version of the signal, but a reflection on an object or surface. In this sense, the work of [21] shows that the difference between both methods is very small.

Regarding the analysis of the signal between dipoles, as can be observed in the results, there is a fairly good correlation between the direct signal and the others obtained from the experiments in air (Figure 11). The best correlation is achieved when the air pulses are inverted, which is consistent with the change of polarity suffered by a propagating pulse that impinges on a metallic object or surface.

The minimum distances between the AUT and the surface to obtain a clear reflection of the latter without interfering with the first have been obtained. In this sense, two approximate separation levels have been established. In the first level (10 dB), the separation is almost complete and there is only an overlap between the components of the pulse of lower energy. From the second level (3dB), the higher energy components begin to overlap and it becomes very difficult to distinguish between the two signals. These distances can be used to get a clear reflection from the surface without aliasing and calculate the permittivity of the surface layer, which in turn leads to the detection of changes in the composition of this layer. Such a methodology has proven to be useful in the study of roads [17, 22]. Here one must also take into account the loss in penetration depth of the antenna in addition to other effects discussed in the section of previous considerations.

To calculate the attenuation curves obtained from the amplitude peaks of the reflected signals in the PEC and metallic bar, the term 1/R^n^, commonly used to characterize the geometric expansion of a wave front, was used. In the case of the PEC, a value of n = 2 has been used, which would be the theoretical value for a spherical wave and a plane reflector of this type (Table 1). It should be remembered that this approach assumes that the object is far enough from the antenna, which explains why the adjustment with this equation is not entirely accurate. The values obtained are completely correlated in both experiments for the 500 MHz antenna. For equation adjustments, values within the overlapping area between the direct signal and the reflection have not been considered as shown in Table 2. However, these amplitude values are used (after application of a moving average filter in two dimensions) to adjust a general exponential regression curve.

As discussed in the methodological section, the aluminium surface used as a reflector in the experiments has a size of 1.80 × 1.80 m. This size is insufficient to contain the entire footprint of the antenna when the antenna is located away from the reflector. In any case, the size of the PEC was chosen so that, for the maximum distance of the experiment (5.80 m), at least the first Fresnel zone of the antenna was contained. At this distance, the radius of the first Fresnel zone will be close to 1.80 m.

**Figure 13.**
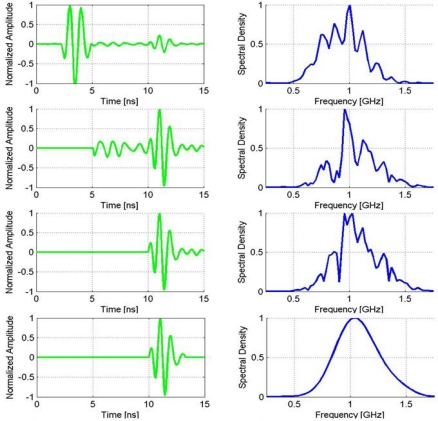
This sequence (from top to bottom) represents the process by which a reflected wavelet is obtained from the entire recorded trace.

When isolating a specific reflected wavelet within the entire recorded trace with the objective of comparing it with other wavelets, it is important to establish a criterion on which parts of the trace can be considered part of this signal. Figure 13 illustrates the problem where a target wavelet is just subsequently cropped tighter.

In Figure 13, the sequence on the left represents the process by which the reflection has been isolated from one of the traces of the experiment on the PEC. The first image in the sequence has little reflection amplitude compared with the direct signal but from the second image is magnified because the direct signal has been erased and the scale self-adjusted to its maximum remaining amplitude. The sequence on the right represents the spectrum associated with the current trace, so one can follow its evolution as the reflection of interest is extracted from the recorded signal.

**Figure 14.**
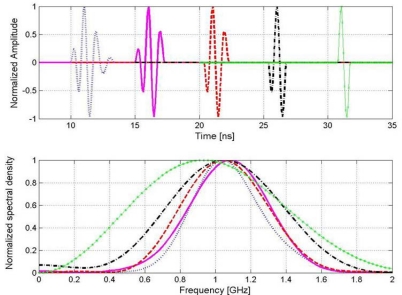
Evolution of the frequency spectrum as the pulse is confined within more stringent limits.

The last graph of the sequence in Figure 13 represents the portion of the signal that seems to be associated with the reflection. The larger peaks are clear and easily distinguishable, but it is not so easy to determine the beginning and end of the pulse since the random noise in the trace has similar amplitude values than the lower peaks of the pulse. In this sense, the fact that the experiments are based not on a single measurement, but on a series of measurements at different distances, helps to clarify which part belongs to the pulse and which is due to the presence of random noise or clutter in a particular trace. Still, when comparing reflections, small changes in the criteria to establish the limits of this signal could give rise to non-negligible variations in the size of the pulse, bandwidth, and central frequency. To illustrate this effect, Figure 14 shows the changes in the frequency spectrum of the pulse obtained in Figure 13 as the pulse is confined within more stringent limits. Thus, when reflected pulses are isolated and compared as in this study, it is important to consider possible contributions of this effect on the results.

Experiments carried out with the buried pipe clarify the antenna behaviour in common materials of interest. There is a decrease of the central frequency and bandwidth associated with a widening of the pulse in time. As discussed before, this is because, first, the medium acts as a low-pass filter, so that the pulse broadens in time narrowing its associated frequency band, and, second, the proximity of the medium makes the antenna frequency spectrum move to lower frequencies. It also highlights the energy loss of the reflection in the metallic bar with respect to the reflection at the PEC for the 500 MHz antenna, possibly due to the low directivity of this antenna.

## Figures and Tables

**Table 1.** Values that allow the best fit of the regression curves and parameters related to the quality of the fit.

	**Type**	**Equation**	**Range**	**a**	**b**	**SSE**	**R-square**

**500 MHz**	Power	*_ax_^b^*	x ≥ 120cm	0.5551	−1.559	0.03609	0.97364
	Exponential	*_ae_^b^*	x ≥ 120cm	1.94	−1.18	0.01572	0.98852
**(M.bar)**	Exponential (all)	*_ae_^b^*	All	1.862	−0.9854	3.29885	0.96995

**500 MHz**	Power	*_ax_^b^*	x ≥ 120cm	0.5551	−1.559	0.03609	0.97364
	Power	*_ax_*^−2^	x ≥ 120cm	1.94	−	0.01572	0.98852
**(PEC)**	Exponential (all)	*_ae_^b^*	All	1.139	−0.172	0.07929	0.9376

**Table 2.** Frequency characteristics of the effective wavelets obtained with the proposed methodologies.

	**f_central_ (air) (MHz)**	**3dB f_L_ (MHz)**	**3dB f_H_ (MHz)**	**BW_3dB_ (MHz)**	**10dB f_L_ (MHz)**	**10dB f_H_ (MHz)**	**BW_10dB_ (MHz)**

**500MHz (Buried pipe)**	425	320	523	230	211	615	404
**500MHz (Metallic bar)**	470	354	581	227	270	864	594
**500MHz (PEC)**	480	605	605	245	267	873	606
